# N-terminal pro-B-type natriuretic peptide level inversely associates with metabolic syndrome in elderly persons

**DOI:** 10.1186/1758-5996-6-15

**Published:** 2014-02-08

**Authors:** Ji-Hung Wang, Chung-Jen Lee, Jen-Che Hsieh, Yu-Chih Chen, Bang-Gee Hsu

**Affiliations:** 1 School of Medicine, Tzu Chi University, Hualien, Taiwan; 2 Division of Cardiology, Buddhist Tzu Chi General Hospital, Hualien, Taiwan; 3 Department of Nursing, Tzu Chi College of Technology, Hualien, Taiwan; 4 Division of Nephrology, Buddhist Tzu Chi General Hospital, No. 707, Section 3, Chung-Yang Road, Hualien, Taiwan

**Keywords:** N-terminal pro-B-type natriuretic peptide, Elderly, Metabolic syndrome

## Abstract

**Aims:**

Serum N-terminal pro-B-type natriuretic peptide (NT-proBNP) was lower in the general population with metabolic syndrome (MetS). The aim of this study was to evaluate the relationship between MetS and fasting serum NT-proBNP concentration in elderly persons.

**Methods:**

Fasting blood samples were obtained from 84 elderly volunteers aged 65 years or older. MetS and its components were defined using diagnostic criteria from the International Diabetes Federation.

**Results:**

Thirty-eight elderly persons (45.2%) had MetS. Fasting NT-proBNP level was negatively correlated with MetS among elderly patients (*p* = 0.001). Univariate linear regression analysis showed that age (*r* = 0.338; *p* = 0.002) was positively correlated with fasting serum log-NT-proBNP levels, while height (*r* = -0.253; *p* = 0.020), body weight (*r* = -0.238; *p* = 0.029), waist circumference (*r* = -0.270; *p* = 0.013), body fat mass (*r* = -0.356; *p* = 0.002) and triglyceride (*r* = -0.291; *p* = 0.007) were negatively correlated with fasting serum log-NT-proBNP levels among the elderly persons. Multivariate forward stepwise linear regression analysis of the significant variables showed that age (*R*^*2*^ change = 0.114, *p* = 0.011), triglyceride (*R*^*2*^ change = 0.118, *p* < 0.001), body fat mass (*R*^*2*^ change = 0.084, *p* < 0.001), and height (*R*^*2*^ change = 0.101, *p* < 0.001) were the independent predictor of fasting serum log-NT-proBNP levels in elderly persons.

**Conclusions:**

NT-proBNP level is significantly reduced in elderly persons affected by MetS, and is significantly positively related to age, while negatively related to triglyceride, body fat mass, height in these subjects.

## Introduction

The heart secretes two natriuretic peptides, atrial natriuretic peptides (ANP) and B-type natriuretic peptides (BNP). They are widely known as body homeostasis regulators that oppose volume expansion [1]. BNP and its N-terminal counterpart, N-terminal pro-B-type natriuretic peptide (NT-proBNP), are cardiac biomarkers that have been established for the assessment of congestive heart failure [2,3]. Recent studies also suggested natriuretic peptides are potent lipolytic agents that act in adipose tissue [4,5]. Low levels of NT-proBNP might lead to reduced lipolysis and excessive weight gain, which may be one of the biological alterations that contribute to the development of metabolic syndrome in the general population (MetS) [6,7].

In old age, fat is redistributed from subcutaneous to intra-abdominal visceral depots as well as other ectopic sites, including bone marrow, muscle and the liver. These changes are associated with increased risk of MetS [8]. MetS increased all-cause and cardiovascular disease mortality in a cohort study of Chinese aged 50 years or older [9]. There is no study about the association between serum NT-proBNP levels and MetS in elderly persons. The aim of this study was to investigate the relationship between the fasting serum NT-proBNP level and MetS among elderly persons.

## Materials and methods

### Participants

Between October 2009 and May 2010, one hundred elderly volunteers aged 65 years or older at a medical center in Hualien, eastern Taiwan were enrolled into this study. The Protection of the Human Subjects Institutional Review Board of Tzu-Chi University and Hospital approved this study. Patients were excluded if they had any acute infection (n = 2), acute myocardial infarction (n = 1), heart failure (n = 5), serum creatinine ≥ 1.2 mg/dl at the time of blood sampling (n = 7) or if they refused to provide informed consent for the study (n = 1). Heart failure defined by the American College of Cardiology Foundation and the American Heart Association 2005 Guidelines. Heart failure is a complex clinical syndrome that can result from any structural or functional cardiac disorder that impairs the ability of the ventricle to fill with or eject blood [10]. Total about eighty-four elderly volunteers (28 males and 56 females) were included in this study.

### Anthropometric analysis

Body weight was measured to the nearest half-kilogram with patients wearing light clothing and without shoes. Height was measured to the nearest 0.5 cm. Waist circumference was measured by tape around patient’s waist from the point between patient’s lowest ribs and patient’s hip bones by placing patient’s hands on patient’s hips. The body mass index (BMI) was calculated as weight (kilograms) divided by height squared (meters). Bioimpedance measurements of fat mass were performed at the bedside according to the standard tetrapolar whole body (hand-foot) technique, using a single-frequency (50-kHz) analyzer (Biodynamic-450, Biodynamics Corporation, Seattle, USA). Measurements were carried out by the same operator. Fat mass was collected and analyzed by specific formulae supplied by the manufacturer. [11,12].

### 2D echocardiographic examination

The 2D echocardiographic recordings were obtained in all patients using commercially available machines. Ejection fraction was calculated by a modification of the method of Quinones et al. [13]. The left ventricular (LV) mass index was calculated as a ratio of LV mass to body surface area. Transmitral flow velocity curves were recorded to measure peak early diastolic (E) and late diastolic (A) velocities. There are three components of the tissue Doppler profile that are routinely measured: the systolic myocardial velocity (S’); the early diastolic myocardial velocity (E’); and the late diastolic myocardial velocity (A’) as previously described [14].

### Biochemical investigations

Fasting blood samples of approximately 5 ml for measuring complete blood count (Sysmex K-1000, Bohemia, NY, USA) and other factors were immediately centrifuged at 3000 *g* for 10 min. Serum levels of blood urea nitrogen (BUN), creatinine (Cre), fasting glucose, total cholesterol (TCH), triglyceride (TG), high-density lipoprotein cholesterol (HDL-cholesterol), and low-density lipoprotein cholesterol (LDL-cholesterol) were measured using an autoanalyzer (COBAS Integra 800, Roche Diagnostics, Basel, Switzerland). Blood samples were assayed for NT-proBNP by electrochemiluminescence immunoassay on the Elecsys 2010 Immunoanalyzer (Roche Diagnostics, Indianapolis, IN, USA).

### Metabolic syndrome and its components

The prevalence of MetS was defined using the International Diabetes Federation definition [15]. People were classified as having MetS if they had central (abdominal) obesity with a waist circumference ≥ 90 cm (men) or ≥ 80 cm (women) (Chinese criteria), and matched two or more of the following criteria: fasting serum glucose of 110 mg/dl or more, triglycerides of 150 mg/dl or higher, HDL-cholesterol level less than 40 mg/dl in men or less than 50 mg/dl in women, or blood pressure of 130/85 mmHg or higher. The use of antihypertensive medication was considered as indicative of high blood pressure in this analysis. Type 2 diabetes was determined according to World Health Organization criteria [16]. A person was regarded as diabetic if the fasting plasma glucose was either 126 mg/dl or more, or if the 2 h glucose during an oral glucose tolerance test was 200 mg/dl or more, or if he/she was using diabetes medication (oral or insulin).

### Statistical analysis

Data are expressed as means ± standard deviation (SD) and were tested for normal distribution by Kolmogorov-Smirnov test. Categorical variables were analyzed by the Chi-square test. Comparisons between patients were performed using the Student’s independent *t* test (two-tailed) for normally distributed data or the Mann–Whitney U test for parameters that presented with non-normal distribution (NT-proBNP). Because the NT-proBNP was non-normal distribution, we used log-NT-proBNP (logarithm of NT-proBNP to base 10) and noted log-NT-proBNP was normal distribution. The significance of differences of NT-proBNP between numbers of metabolic syndrome criteria was analyzed by the Kruskal-Wallis analysis of variance (AVONA) test. Clinical variables that correlated with serum log-NT-proBNP levels in elderly persons were evaluated by univariate linear regression analyses. Variables that were significantly associated with log-NT-proBNP in elderly persons were tested for independency in multivariate forward stepwise regression analysis. Data were analyzed using SPSS for Windows (version 13.0; SPSS Inc., Chicago, IL, USA). A *p*-value of less than 0.05 was considered statistically significant.

## Results

The clinical, echocardiographic recordings and laboratory characteristics of the elderly persons are presented in Table 1 and Table 2. Medical histories included: diabetes (n = 30; 35.7%), and hypertension (n = 68; 80.9%). The use of drugs included: angiotensin receptor blocker (ARB; n = 40; 47.4%), angiotensin-converting enzyme inhibitor (ACEI; n = 16; 19.1%), calcium channel blocker (CCB; n = 46; 54.8%), β-blocker (n = 38; 45.2%), statin (n = 20; 23.8%), fibrate (n = 10; 11.9%), sulfonylurea (n = 8; 9.5%), and metformin (n = 18; 21.4%). Thirty-eight patients (45.2%) had MetS, whereas 46 patients (54.8%) did not. Elderly persons who had MetS had lower serum fasting NT-proBNP levels than those without MetS (*p* = 0.001). NT-proBNP levels did not differ statistically by gender distribution, diabetes, hypertension, ARB, ACEI, CCB, β-blocker, statin, fibrate, sulfonylurea, or metformin drugs used.

**Table 1:** Clinical and analytical characteristics of 84 elderly persons

**Item**	**Parameter**		**Parameter**	
Anthropometric findings	Height (cm)	156.89 ± 6.00	Waist circumference (cm)	96.02 ± 9.68
	Body weight (kg)	67.53 ± 9.86	Body fat mass (%)	37.31 ± 7.14
	Body mass index (kg/m^2^)	27.40 ± 3.53	Age (year)	73.31 ± 4.66
	DBP (mmHg)	72.53 ± 7.36	SBP (mmHg)	131.96 ± 9.86
Echocardiographic finding	LV ejection fraction (%)	66.59 ± 13.47	LV mass index (g/m2)	103.37 ± 26.64
	Mitral E (cm/s)	51.85 ± 17.11	Mitral A (cm/s)	80.89 ± 20.20
	Mitral E/A ratio	0.67 ± 0.28	E’ (cm/s)	5.87 ± 1.98
	A’ (cm/s)	10.48 ± 2.67	E/E’ ratio	9.73 ± 5.86
	S’ (cm/s)	7.90 ± 1.57		
Biochemical findings	Triglyceride (mg/dl)	142.30 ± 82.39	Total cholesterol (mg/dl)	192.60 ± 37.74
	Fasting glucose (mg/dl)	114.16 ± 29.83	HDL-cholesterol (mg/dl)	49.69 ± 13.34
	LDL-cholesterol (mg/dl)	125.58 ± 35.26	Creatinine (mg/dl)	0.87 ± 0.17
	BUN (mg/dl)	17.61 ± 4.77	WBC (x1000/ul)	6.85 ± 1.69
	Hemoglobin (g/dl)	13.54 ± 1.84	NT-proBNP (pg/ml)	251.26 ± 282.69

Data are expressed as means ± standard deviations or proportion.

SBP, systolic blood pressure; DBP, diastolic blood pressure; LV, left ventricular; A’, late diastolic myocardial velocity; E’, early diastolic myocardial velocity; S’, systolic myocardial velocity; E/E’, ratio of peak mitral E wave velocity to peak early diastolic myocardial velocity at septal position recorded by tissue Doppler imaging; HDL-cholesterol, high-density lipoprotein cholesterol; LDL-cholesterol, low-density lipoprotein cholesterol; BUN, blood urea nitrogen; WBC, white blood cells; NT-proBNP, N-terminal pro-B-type natriuretic peptide.

**Table 2:** Clinical characteristics and fasting serum N-terminal pro-B-type natriuretic peptide levels of 84 elderly persons

**Characteristic**		**Number (%)**	**NT-proBNP (pg/ml)**	***P *** **value**
Gender	Male	28 (33.3)	173.50 ± 227.45	0.284
	Female	56 (66.7)	290.14 ± 302.88	
Diabetes	No	54 (64.3)	218.80 ± 275.99	0.636
	Yes	30 (35.7)	269.30 ± 289.92	
Hypertension	No	16 (19.1)	336.25 ± 370.62	0.223
	Yes	68 (80.9)	231.26 ± 260.74	
Metabolic syndrome	No	46 (54.8)	384.87 ± 342.22	0.001*
	Yes	38 (45.2)	89.53 ± 55.88	
ACE inhibitor	No	68 (80.9)	236.53 ± 281.99	0.748
	Yes	16 (19.1)	313.88 ± 296.03	
ARB	No	40 (47.6)	306.30 ± 315.87	0.251
	Yes	44 (52.4)	201.23 ± 245.44	
β-blocker	No	46 (54.8)	262.09 ± 310.44	0.622
	Yes	38 (45.2)	238.16 ± 252.79	
CCB	No	38 (45.2)	326.26 ± 325.47	0.100
	Yes	46 (54.8)	189.30 ± 231.05	
Statin	No	64 (76.2)	276.50 ± 306.19	0.506
	Yes	20 (23.8)	170.50 ± 177.76	
Fibrate	No	74 (88.1)	264.46 ± 296.72	0.938
	Yes	10 (11.9)	168.40 ± 130.17	
Sulfonylurea	No	76 (90.5)	265.50 ± 293.33	0.780
	Yes	8 (9.5)	116.00 ± 62.79	
Metformin	No	66 (78.6)	249.15 ± 268.06	0.690
	Yes	18 (21.4)	259.00 ± 349.32	

Data are expressed as means ± standard deviations.

*P < 0.05 was considered statistically significant after Mann–Whitney U test.

NT-pro-BNP, N-terminal pro-B-type natriuretic peptide; ARB, angiotensin-receptor blocker; ACE, angiotensin-converting enzyme; CCB, calcium-channel blocker.

**Figure 1:**
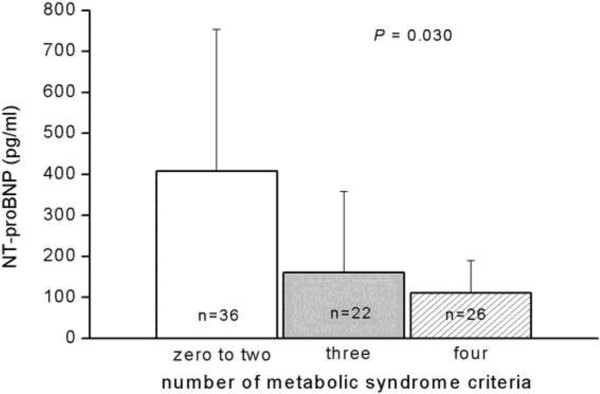
**Fasting serum N-terminal pro-B-type natriuretic peptide levels in different metabolic syndrome diagnostic criteria among the 84 elderly persons.** Data was analyzed by the Kruskal-Wallis analysis of variance (AVONA) test.

Fasting serum NT-proBNP levels in different metabolic syndrome diagnostic criteria are presented in Figure 1. There was a tendency for decreased fasting NT-proBNP levels as the number of diagnostic criteria for metabolic syndrome in patients increased. A statistically significant difference between the number of metabolic syndrome criteria and serum NT-proBNP levels in elderly persons (*p* = 0.030) was established.

The univariate linear analysis of fasting serum log-NT-proBNP levels in elderly persons is presented in Table 3. Ages (*r* = 0.338; *p* = 0.002) was positively correlated with fasting serum log-NT-proBNP levels, while height (*r* = -0.253; *p* = 0.020), body weight (*r* = -0.238; *p* = 0.029), waist circumference (*r* = -0.270; *p* = 0.013), body fat mass (*r* = -0.356; *p* = 0.002) and triglyceride (*r* = -0.291; *p* = 0.007) were negatively correlated with fasting serum log-NT-proBNP levels among the elderly persons (Table 3).

**Table 3:** Correlation of logarithm of fasting serum N-terminal pro-B-type natriuretic peptide levels and clinical variables by univariate linear regression analyses among the 84 elderly persons

**Items**	**Beta**	***P *** **value**
Age (year)	0.338	0.002*
Height (cm)	-0.253	0.020*
Body weight (kg)	-0.238	0.029*
Waist circumference (cm)	-0.270	0.013*
Body mass index (BMI; kg/m^2^)	-0.140	0.205
Body fat mass (%)	-0.326	0.002*
White blood cell (x1000/ul)	-0.070	0.544
Hemoglobin (g/dl)	-0.107	0.144
Total cholesterol (mg/dl)	-0.016	0.888
Triglyceride (mg/dl)	-0.291	0.007*
HDL-C (mg/dl)	0.188	0.087
LDL-C (mg/dl)	0.013	0.908
Fasting glucose (mg/dl)	0.210	0.056
Blood urea nitrogen (mg/dl)	0.163	0.144
Creatinine (mg/dl)	-0.133	0.227
Systolic pressure (mmHg)	0.103	0.351
Diastolic pressure (mmHg)	-0.110	0.317

*P < 0.05 was considered statistically significant after univariate linear analyses.

HDL-cholesterol, high-density lipoprotein cholesterol; LDL-cholesterol, low-density lipoprotein cholesterol.

**Table 4:** Multivariate stepwise linear regression analysis of age, height, body weight, waist circumference, body fat mass, and triglyceride: correlation to logarithm of fasting serum N-terminal pro-B-type natriuretic peptide levels among 84 elderly persons

**Items**	**Beta**	**R square**	**R square change**	***P *** **value**
Age (year)	0.242	0.114	0.114	0.011*
Triglyceride (mg/dl)	-0.392	0.232	0.118	< 0.001*
Body fat mass (%)	-0.398	0.316	0.084	< 0.001*
Height (cm)	-0.362	0.417	0.101	< 0.001*

*P <0.05 was considered statistically significant after multivariate stepwise linear regression analyses.

Multivariate forward stepwise linear regression analysis of the variables that were significantly associated with fasting serum log-NT-proBNP levels among elderly persons showed that age (*β* = 0.242, *R*^*2*^ change = 0.114, *p* = 0.011), triglyceride (*β* = -0.392, *R*^*2*^ change = 0.118, *p* < 0.001), body fat mass (*β* = -0.398, *R*^*2*^ change = 0.084, *p* < 0.001), and height (*β* = -0.362, *R*^*2*^ change = 0.101, *p* < 0.001) were the independent predictor of fasting serum log-NT-pro-BNP levels (Table 4).

## Discussion

The results of our study showed that the fasting NT-proBNP level was negatively associated with MetS in elderly persons, and that age, triglyceride, body fat mass and height were independent predictors of fasting serum NT-proBNP levels.

In elderly people disorders occur that are closely related to the ageing process. They include symptoms from the cardiovascular system, such as elevated systolic blood pressure, pulse pressure, increased mass of the left ventricle and more frequent ischemic heart disease, as well as cardiac arrhythmias, and particularly atrial fibrillation [17]. As compared to normal volunteers echocardiographic data at Aizawa et al. [14] our study noted the E/A ratio and E’ were lower, and the LV mass index was higher in elderly persns.

MetS is a constellation of cardiovascular risk factors that has been associated with increased risk of cardiovascular disease, diabetes, and mortality as well as other adverse health outcomes [15]. Aging is characterized by body fat redistribution with increased visceral fat and relative loss of subcutaneous fat, especially in the periphery. The impaired capacity of fat tissue to store lipids associated with aging may be responsible for increased systemic free fatty acid exposure, leading to ectopic fat deposition, lipotoxicity and metabolic disease [8]. Anthropometric changes with an increase in fat mass with a parallel decline in fat free mass, environmental changes such as dietary habit changes and reduced physical activity, neurohormonal variations which may have an opposing effect on insulin, and an increase in oxidative stress in the elderly induce insulin resistance and are associated with impaired glucose handling, mainly through the decline of insulin action in the elderly [18]. The MetS is associated with the incidence of diabetes, and the synergy between MetS and diabetes is an important risk factor for all-cause mortality in elderly subjects [19]. MetS in an elderly population is a proven risk factor for all-cause and cardiovascular disease mortality [9]. The prevalence of MetS in the United States is 43.5% and 42.0% for participants aged 60 through 69 years and aged at least 70 years, respectively [20]. Our study noted the overall prevalence of MetS is 45.2% in the elderly and the prevalence of MetS was 42.9% (12/28) in men and 46.4% (26/56) in women.

The renin-angiotensin-aldosterone system is activated in obese patients and angiotensin II acting as a ‘growth factor’ for adipose tissue growth and development [4]. BNP binds its common receptor, guanylyl cyclase-A (GC-A), which leads to biological actions through a cyclic guanosine monophosphate (cGMP)-dependent pathway and has an inhibition effect of the renin–angiotensin–aldosterone axis [2]. So, low levels of natriuretic peptide may activate the renin-angiotensin-aldosterone system, which may contribute to the development of obesity. In normal subjects, natriuretic peptide may affect the homoeostasis of glucose and lipid metabolism, partly through the reduction in adipogenesis, as well as the increased release and more efficient consumption of non-esterified fatty acids by peripheral tissues [21]. It can be speculated that low levels of natriuretic peptide may lead to reduced lipolysis and excessive weight gain, which may be one of the biological alterations that contribute to the development of obesity [5]. BNP through a cGMP-dependent pathway can promote muscle mitochondrial biogenesis and fat oxidation, as to prevent obesity and glucose intolerance in mice [22]. Some studies did not find an association between serum NT-proBNP concentrations and MetS [23,24]. However, other studies noted serum NT-proBNP was lower in the general population with MetS [6,7]. Recent study on elderly population from Poland noted obese patients had significantly more frequently NT-proBNP values < 400 pg/ml (73.0%) and less frequently NT-proBNP values > 2000 pg/ml (2.8%) [25]. Our study also noted that elderly persons with MetS had lower fasting serum NT-proBNP levels. Moreover, our study also noted a tendency for decreasing fasting serum NT-proBNP levels as the number of diagnostic criteria for metabolic syndrome in elderly persons increased.

NT-proBNP values were substantially higher in women compared to men at every age, and levels increased with increasing age for both genders in healthy individuals from the Framingham Heart Study Generation 3 cohort study [26]. This study also noted upper reference limit for NT-proBNP implied is more than 2-fold higher in women than men [26]. Our result also showed that age was positively correlated with fasting serum log-NT-proBNP levels and nearly 2-fold higher in women than men among the elderly persons. Although gender differences in circulating natriuretic peptides have been previously reported, the underlying mechanisms remain unclear [27]. In symptomatic left ventricular systolic heart failure patients a higher BMI is associated with decreased NT-proBNP levels [28]. Our study noted that height and body weight were negatively correlated with fasting serum log-NT-proBNP levels in elder persons, but did not differ statistically by BMI in this study. Plasma NT-proBNP was inversely associated with visceral adipose tissue volumes measured by multidetector computed tomography in a study of 1,873 community-based individuals [29]. However, another study noted NT-proBNP was inversely associated with hematocrit and hepatic steatosis, while no association was found with waist circumference and skinfold fat measurement in the elderly [30]. Serum NT-proBNP was inversely related to triglyceride [6,24]. Our study also showed that waist circumference, body fat mass, and triglyceride were negatively correlated with fasting serum log-NT-proBNP levels among the elderly persons. Multivariate forward stepwise linear regression analysis of significant variables showed that age, triglyceride, body fat mass, and height were waist circumference was the independent predictor of fasting serum log-NT-proBNP levels in our study.

Pharmacological interventions have been shown to influence serum NT-proBNP in humans. The use of valsartan reduces plasma concentrations of NT-proBNP in post–myocardial infarction and congestive heart failure patients [31]. After 8 weeks of treatment with losartan a significant decrease was noted in the levels of NT-proBNP in patients after acute myocardial infarction [32]. NT-proBNP was significantly reduced after six months of treatment with enalapril or carvedilol combined with enalapril treatment in stable systolic heart failure patients. In contrast, no change was observed in the carvedilol group [33]. Atorvastatin treatment reduced NT-proBNP levels in heart failure patients [34]. Our results did not show a relationship between statins or other drugs (angiotensin receptor blocker, angiotensin-converting enzyme inhibitor, calcium channel blocker, or β-blocker) and serum NT-proBNP among elderly persons. Further studies are required to elucidate the relationship between medication and NT-proBNP in elderly persons.

Our study has some limitations. First, the number of patients enrolled was small and there was weak statistical power in terms of the small number of patients. Secondly, this study had a cross-sectional design. NT-proBNP, have been shown a high prognostic value in total mortality, cardiovascular mortality in the elderly population and is independent predictor of all-cause mortality in patients with acute heart failure at 30 days and 1 year after emergency department in elderly persons [35,36]. Therefore, our findings should be investigated in long-term prospective studies before a causal relationship between serum NT-proBNP and MetS in elderly persons can be established. It should also be noted that BNP can enhance adiponectin production by human adipocytes in vitro and even in patients with heart failure [37]. Serum adiponectin values were found to correlate inversely with the presence of MetS [38]. Adiponectin is independently associated with NT-proBNP in the general population [39] and in elderly coronary artery disease patients [40]. Additional studies are required to ascertain whether the serum concentration of adiponectin is associated with NT-proBNP in elderly persons.

## Conclusion

Low levels of NT-proBNP might lead to reduced lipolysis and excessive weight gain, which may be one of the biological alterations that contribute to the development of MetS. The present study shows a negative association between circulating fasting NT-proBNP and MetS among elderly persons. Age, triglyceride, body fat mass and height were independent predictor of the serum log-NT-proBNP level among elderly persons.

## Abbreviations

ANP: Atrial natriuretic peptides; BNP: B-type natriuretic peptides; NT-proBNP: N-terminal pro-B-type natriuretic peptide; MetS: Metabolic syndrome; BMI: Body mass index; BUN: Blood urea nitrogen; Cre: Creatinine; HDL-C: High-density lipoprotein-cholesterol; LDL-C: Low-density lipoprotein-cholesterol; TCH: Total cholesterol; TG: Triglyceride; GC-A: Guanylyl cyclase-A; cGMP: Cyclic guanosine monophosphate.

## Competing interests

All authors declare that they have no competing interests.

## Authors’ contributions

LCJ researched and analyzed data. WJH and HBG designed of the study, interpretation of data and wrote the manuscript. HJC, CYC and WJH collected the data. All authors read and approved the final version of the manuscript.
